# Biogenic synthesis of Zinc oxide nanostructures from *Nigella sativa* seed: Prospective role as food packaging material inhibiting broad-spectrum quorum sensing and biofilm

**DOI:** 10.1038/srep36761

**Published:** 2016-12-05

**Authors:** Nasser A. Al-Shabib, Fohad Mabood Husain, Faheem Ahmed, Rais Ahmad Khan, Iqbal Ahmad, Edreese Alsharaeh, Mohd Shahnawaz Khan, Afzal Hussain, Md Tabish Rehman, Mohammad Yusuf, Iftekhar Hassan, Javed Masood Khan, Ghulam Md Ashraf, Ali Mohammed Alsalme, Mohamed F. Al-Ajmi, Vadim V. Tarasov, Gjumrakch Aliev

**Affiliations:** 1 Department of Food Science and Nutrition, College of Food and Agriculture, King Saud University, Riyadh-11451, Kingdom of Saudi Arabia; 2 College of Science & General Studies, Alfaisal University, Riyadh 11533, Kingdom of Saudi Arabia; 3 Department of Chemistry, College of Science, King Saud University, Riyadh-11451, Kingdom of Saudi Arabia; 4 Department of Agricultural Microbiology, Aligarh Muslim University, Aligarh-202002, India; 5 Protein Research Chair, Department of Biochemistry, College of Science, King Saud University, Riyadh-11451, Kingdom of Saudi Arabia; 6 Department of Pharmacognosy, College of Pharmacy, King Saud University, Riyadh-11451, Kingdom of Saudi Arabia; 7 Department of Botany, Aligarh Muslim University, Aligarh-202002, India; 8 Department of Zoology, College of Sciences, King Saud University, Riyadh, Saudi Arabia; 9 King Fahd Medical Research Center, King Abdulaziz University, Jeddah, Saudi Arabia; 10 Institute of Pharmacy and Translational Medicine, Sechenov First Moscow State Medical University, 2-4 Bolshaya Pirogovskaya St., 119991 Moscow, Russia; 11 GALLY International Biomedical Research Consulting LLC., 7733 Louis Pasteur Drive, #330, San Antonio, TX, 78229, USA; 12 School of Health Science and Healthcare Administration, University of Atlanta, E. Johns Crossing, #175, Johns Creek, GA, 30097, USA; 13 Institute of Physiologically Active Compounds Russian Academy of Sciences, Chernogolovka, 142432, Russia

## Abstract

Bacterial spoilage of food products is regulated by density dependent communication system called quorum sensing (QS). QS control biofilm formation in numerous food pathogens and Biofilms formed on food surfaces act as carriers of bacterial contamination leading to spoilage of food and health hazards. Agents inhibiting or interfering with bacterial QS and biofilm are gaining importance as a novel class of next-generation food preservatives/packaging material. In the present study, Zinc nanostructures were synthesised using *Nigella sativa* seed extract (NS-ZnNPs). Synthesized nanostructures were characterized hexagonal wurtzite structure of size ~24 nm by UV-visible, XRD, FTIR and TEM. NS-ZnNPs demonstrated broad-spectrum QS inhibition in *C. violaceum* and *P. aeruginosa* biosensor strains. Synthesized nanostructures inhibited QS regulated functions of *C. violaceum* CVO26 (violacein) and elastase, protease, pyocyanin and alginate production in PAO1 significantly. NS-ZnNPs at sub-inhibitory concentrations inhibited the biofilm formation of four-food pathogens viz. *C. violaceum* 12472, PAO1, *L. monocytogenes, E. coli*. Moreover, NS-ZnNPs was found effective in inhibiting pre-formed mature biofilms of the four pathogens. Therefore, the broad-spectrum inhibition of QS and biofilm by biogenic Zinc oxide nanoparticles and it is envisaged that these nontoxic bioactive nanostructures can be used as food packaging material and/or as food preservative.

Major problem concerning the food industry is microbial spoilage of food and severe economic losses are incurred as result of microbial spoilage and/or contamination of food items with pathogens^1^. Quorum sensing (QS), a bacterial cell communication system is often associated with the bacterial spoilage of food products. Considering the importance of QS in food microbial ecology, development of novel food preservatives, packing materials that can specifically block QS and prevent losses due to spoilage of food is the need of the hour^2^,^3^. Quorum sensing or cell-to-cell communication between bacteria commonly associated with contamination of food takes place by the production of signaling molecules called autoinducers and this bacterial cross talk can be intra as well inter species specific^4^. Quorum sensing is known to regulate several proteolytic, lipolytic, chitinolytic, and pectinolytic activities associated with the deterioration of foods. Moreover, several types of signaling molecules have been detected in different spoiled food products^2^. Hence, disruption of quorum-sensing circuit has been identified as one of the prime targets to control microbial gene expression associated with food spoilage and subsequent infection. There is an urgent need to understand the role of quorum-sensing signaling molecules involved in food spoilage and develop novel, safe QS inhibitors that can interfere with bacterial signaling system and prevent food spoilage and biofilm formation by food-related bacteria.

Cell to cell communication (QS) occurs across both Gram-positive as well as Gram-negative bacteria. Three major types of autoinducers have been recognized: acyl-homoserine lactones (AHLs), autoinducing peptides (AIPs) and autoinducer-2 (AI-2s) molecules^5^. These signal molecules regulate the production of various functions like pectinase, protease, siderophore-mediated iron chelation, characteristics associated with food spoilage^2^. AHL based QS regulates the production of violacein pigment in *Chromobacterium violaceum*, virulence in *Pseudomonas aeruginosa*, flagellar motility in *Listeria monocytogenes*, bioluminescence in *Vibrio harveyi* and *V. fischeri*, sporulation, cell differentiation and community organization which lead to the development of the mature biofilms^6^,^7^.

Numerous studies have linked QS to biofilm formation in food-related bacteria. QS plays a key role in all the stages of biofilm formation including the initial attachment of the bacteria to the maturation of the biofilm^3^. Biofilms formed on food surfaces are a major problem area as they act as carriers of bacterial contamination leading to spoilage of food and health hazards^8^. Eradication of biofilms from food contact surfaces and other equipments is difficult and has attracted the attention of the scientific community worldwide^3^,^9^.

Compounds inhibiting or interfering with bacterial QS and biofilm are gaining importance as a novel class of next-generation food preservatives as well as antimicrobial agents^10^. QS inhibitors target the virulence mechanism of the bacteria without inhibiting its growth. Hence, reducing the chances of development of resistance as no selective pressure is exerted on the pathogen^11^,^12^.

Potential of nanotechnology has revolutionized the food packaging industry and safe packaging material with improved properties have developed. Further, integration of antimicrobial agents, antioxidants, and nanosensors for monitoring the quality of food are expected to provide advanced packaging solutions^13^,^14^. Nanoparticles (NPs) of metals and their oxides are being exploited by the industrial sector and several medical; pharmaceutical applications are well known^15^. Synthesis of NPs has been reported using various chemical and physical methods^16^ but recently biological synthesis has gained importance because of the rapidity, safety, stability, and economical attributes associated with the method. This biogenic green synthesis approach involves the biomolecules such as proteins, amino acids, enzymes, vitamins, alkaloids, phenolics, saponins, tannins, and terpinoids, present in plant extracts, for reduction and stabilization of metal ions^17^.

This has prompted us to use *Nigella sativa* (NS) plant extract for synthesis of zinc oxide nanoparticles (ZnONPs). Zinc oxide has been chosen because of its wide spectrum applications in cosmetics, paints, plastic and rubber manufacturing, pharmaceutical products, diagnostics and microelectronics^18^. The ZnONPs have also been used in heavy metal removal from water^19^, and in dental applications^20^. They have also been shown to exhibit strong protein adsorption properties, which can be used to modulate cytotoxicity, metabolism and other cellular responses^21^. Also, due to its low toxicity, ZnO has been listed as “Generally Recognized as Safe” (GRAS) by the US Food and Drug Administration (21, CFR 182, 8991). Therefore, *N. sativa*seed extract has been used to synthesize NS-ZnNPs, by reduction of ZnNO_3_ without involving any supplementary chemicals. These seeds are used as spice, food additive and as a preservative^22^. It exhibits wide pharmacological properties such as diuretic, antihypertensive, antidiabetic, anticancer and anti-inflammatory properties^23^. Previously, extract of this plant has been used for synthesis of silver NPs (~4–17 nm) and gold NPs (~12–20 nm)^24^,^25^,^26^.

This study synthesizes the zinc oxide nanoparticles for the first time from the seed extract of *N. sativa*. Present investigation focusses on the development of synthesized NS-ZnNPs as inhibitors of quorum sensing and its regulated functions in food borne pathogens. Besides, the biofilm inhibitory properties of the biogenic nanoparticles are also explored against pathogens such as *Listeria monocytogenes, Pseudomonas aeruginosa, E. coli* and *Chromobacterium violaceum* for potential use as food preservative and active food packaging material ensuring food safety.

## Material and Methods

### Bacterial strains and growth conditions

*C. violaceum* 12472 is a wild-type strain that produces a QS regulated purple coloured pigment, violacein in response to cognate C_4_ and C_6_ Acyl homoserine lactone molecules. *Chromobacterium violaceum* CVO26 is a Tn5 mutant strain that only produces violacein when short-chain autoinducers are added. *P. aeruginosa* PAO1 is an opportunistic pathogenic bacteria and many of its virulence factors and traits are QS controlled^27^. *Escherichia coli, Listeria monocytogenes* used in study are laboratory strains. All strains were maintained on Luria Bertani or LB broth (15.0 g tryptone, 0.5% yeast extract, 0.5% NaCl) solidified with 1.5% agar (Hi-media). *C. violaceum* 12472, *C. violaceum* CVO26 and *P. aeruginosa* PAO1 strains were cultivated at 28 °C and 37 °C respectively.

All the analytical grade chemicals were procured without further purification from the standard brand of E. Merck Limited, India. The common solvent medium used throughout the synthesis was double distilled water (DDW). The glassware’s utilized during the reactions were received from Borosil, India.

### Preparation of *Nigella sativa* (NS) seed extract

*Nigella sativa* seed extract was prepared by crushing seed in a grinder. Resultant seed powder was thoroughly washed in distilled water and 10 g of this powder was homogenized completely in 50 ml double distilled water and incubated with constant stirring (100 rpm) at 80 °C for 20 min. The resultant mixture then filtered using Whatman filter papers No. 1 to remove debris. This extract was used for generating green zinc nanoparticles. The extract was stored at 4 °C for future uses.

### Zinc nanoparticle synthesis

All of the reagents involved in the experiments were of analytical grade purity and utilized as received without further purification. Zinc nitrate (99.999%) was purchased from Sigma Aldrich, and the seed extract was collected. The synthesis was carried out in a domestic microwave oven (Samsung, 750 W). In a typical experiment, a 0.05 M aqueous solution of zinc nitrate in 100 ml distilled water was prepared in which 10 ml *Nigella sativa* seed extract was added to obtain a mixture solution in a round-bottom flask, and then put into a domestic microwave oven. Microwave irradiation proceeded at 100% power for 20 min. After microwave processing, the solution was cooled to room temperature. The resulted precipitate was separated by centrifugation, then washed with deionized water and absolute ethanol for several times, and dried in an oven at 80 °C for 24 h. Finally, the product was calcined at 800 °C for 2 h.

### Characterization

The phase purity of the as-obtained product was characterized by X-ray diffraction using Rigaku (Miniflex-2) X-ray diffractometer with Cu Kα radiations (λ = 1.5406 Å) operated at voltage of 40 kV and current of 15 mA. The particle size and surface morphology of the synthesized powders were carried out using field emission transmission electron microscope (FE-TEM; JEOL/JEM-2100F version) operated at 200 kV. Fourier transmission infrared (FTIR) spectra of the powders (as pellets in KBr) were recorded using a Fourier transmission infrared spectrometer (Perkin Elmer) in the range of 4000–400 cm^−1^ with a resolution of 1 cm^−1^. Room temperature optical absorption spectrum was recorded in the range of 200–800 nm using a UV-Vis spectrophotometer (Perkin Elmer). The thermal study of the synthesized product was carried out using thermo gravimetric analyzer (TGA-DTA; Thermo Scientific) up to 1000 °C in air at the heating rate of 10 °C/min, after purging N_2_ gas.

### Screening for anti-QS activity

A standard disc diffusion assay was used to determine the anti-QS activity of NS-ZnNPs^28^. Overnight grown culture of *C. violaceum* 12472 (100 μl) was mixed with 5 mL of molten LB agar (0.3%) and immediately poured over the surface of pre-poured LB agar plate. After solidification, sterilized paper discs were placed on the surface of medium. Various concentrations of the synthesized NS-ZnNPs were applied on the discs and incubated overnight at 30 °C. Zone of inhibition of violacein pigment around the disc was considered as anti-QS activity.

### Determination of Minimum inhibitory concentration (MIC)

The MICs of synthesized NS-ZnNPs against the bacterial pathogens were determined using the CLSI macrobroth dilution method^29^. MIC is defined as the minimum concentration of ZNPs at which there was no visible growth of the test strains. Concentrations below the MICs were considered sub-inhibitory and were further used to study the anti-QS and biofilm inhibitory properties.

### Violacein inhibition assay

Biosensor strain *Chromobacterium violaceum* CV026 was incubated for 16–18 h (OD_600 nm_ = 0.1) and inoculated to in Erlenmeyer flasks containing Luria broth (LB), LB supplemented with C6-HSL (10 μM/l) and LB supplemented with C6-HSL and test agent. The flasks were incubated at 27 °C with 150 rev/min agitation for 24 h in a shaking incubator^30^.

Violacein production by *Chromobacterium violaceum* (CVO26) in presence of NS-ZnNPs was studied using method described by Husain *et al*.^30^.

### Effect on virulence factors production

Effect of Sub-MICs of NS-ZnNPs on virulence factors of *P. aeruginosa* such as LasB elastase, protease, pyocyanin, alginate production was determined using protocols described previously^31^,^32^.

### Analysis of *lasB and pqsA* transcriptional activity in *E. coli*

*lasB and pqsA* transcriptional activity in *E. coli* MG4/pKDT17 and *E. coli* pEAL08-2 in was measured using the β-galactosidase assay described by Pearson *et al*.^33^ and Cugini *et al*.^34^.

### Swarming motility assay

Swarming motility was determined as described earlier^28^. Briefly, overnight culture of test pathogens was point inoculated at the center of the medium consisting of 1% tryptone, 0.5% NaCl and 0.3% agar with or without sub-MICs of synthesised NS-ZnNPs.

### Extraction and quantification of exopolysaccharide (EPS)

Test strains of *P. aeruginosa, E. coli, L. monocytogenes*, and *C. violaceum* were grown in the presence and absence of sub-MICs of synthesised NS-ZnNPs were centrifuged and the resulting supernatant was filtered. Three volumes of chilled 100% ethanol were added to the filtered supernatant and incubated overnight at 4 °C to precipitate EPS^35^. EPS was then quantified by measuring sugars following the method of Dubois *et al*.^36^.

### Assay for biofilm inhibition

The effect of NS-ZnNPs on biofilm formation was measured using the polyvinyl chloride biofilm formation assay^37^. Briefly, overnight cultures of test pathogens were re-suspended in fresh LB medium in the presence and the absence of NS-ZnNPs and incubated at 30 °C for 24 h. The biofilms in the microtiter plates stained with a crystal violet solution and quantified by solubilizing the dye in ethanol and measuring the absorbance at OD_470_.

### *In situ* visualization of the biofilms

Briefly, 1% of overnight cultures of the test pathogens (0.4 OD at 600 nm) were added into 1 ml of fresh LB medium containing cover glass of 1 cm^2^ along with and without NS-ZnNPs at respective sub-MICs. After 24 h of incubation, the cover glasses were rinsed thrice with distilled water to remove the planktonic cells and biofilms in the cover glasses were stained with 0.2% crystal violet (CV) solution. Stained cover glasses were placed on slides with the biofilm pointing up and visible biofilms were visualized by light microscope at magnifications of 40X (Nikon Eclipse Ti 100, Japan).

For CLSM analysis, biofilms of various bacterial strains were allowed to form on the glass coverslips as described in light microscopic analysis. After 24 h, biofilm formed in the glass slides were stained with 20 μl of 1% acridine orange (Sigma Aldrich, Switzerland). The excess stain was washed out and the stained cover glasses were visualized with CLSM (Zeiss Spinning disk confocal microscope, Zeiss, Germany) equipped with an excitation filter 515–560 and magnification at 40×.

### Inhibition of mature biofilms

To ensure the potential of NS-ZnNPs in disturbing the mature biofilm of pathogenic bacterial organisms (*P. aeruginosa, E. coli, L. monocytogenes*, and *C. violaceum*), biofilms of bacterial pathogens were initially allowed to develop in microtitre plates for 16 h. Mature biofilms were then treated with respective sub-MICs of NS-ZnNPs for 8 h, stained with a crystal violet and quantified by solubilizing the dye in ethanol and measuring the absorbance at OD_470_.

### Antibacterial activity of NS-ZnNPs in food model media

Lettuce leaf model media (L) was prepared as described by Gutierrez *et al*.^38^ but with some modifications. 50 g of lettuce (*Lactuca sativa* sp.) were added to 100 ml of sterile deionized water and shaken for 1 min. The suspension was filtered using 18.5 cm Whatman filters and pH was adjusted from 5.6 to 7.2 by mixing two parts of lettuce media with one part 0.3 M potassium phosphate buffer, giving a final concentration of 0.1 M phosphate buffer, pH 7.2. The buffered medium was then autoclaved at 121 °C for 15 min. To investigate the NS-ZnNPs efficacy in meat-based model media, experiments were performed with autoclaved beef extract (BE, 12% protein). Beef extract model media was adjusted to pH 7.2 to rule out pH effects.

The effect of NS-ZnNPs (2 × MIC) on the growth of *P. aeruginosa, E. coli, L. monocytogenes*, and *C. violaceum* in Lettuce leaf and beef extract food model media was assessed by optical density measurements. Bacteria were grown in NS-ZnNPs treated lettuce leaf model media, beef extract media, and growth was monitored in a microplate reader (Thermo Scientific Multiskan FC) at 600 nm over 24 h at 4 h intervals.

### Statistical analysis

All experiments were performed in triplicate and the data obtained from the experiments were presented as mean values and the differences between control and test were analyzed using Student’s *t* test.

## Results

**Figure 1:**
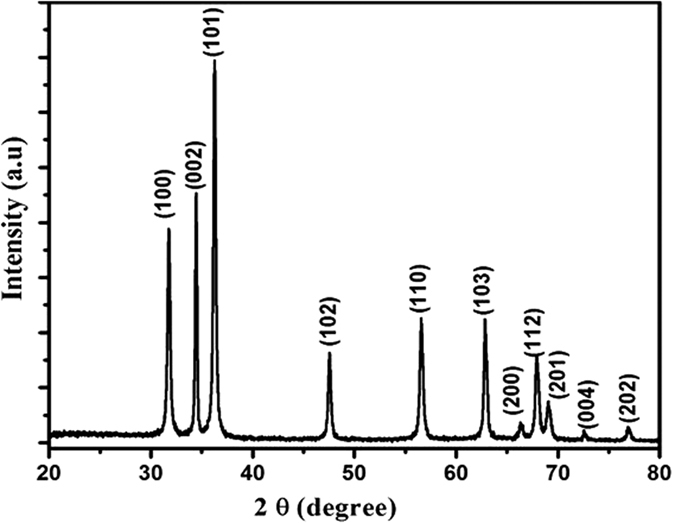
XRD patterns of ZnO nanoparticles using *Nigella sativa* seed extract.

XRD measurements were performed to determine the crystalline phase of the samples. Figure 1 shows the XRD patterns of ZnO sample, which was indexed using POWDER-X software as the ZnO wurtzite structure and well matched with the standard data (JCPDS, 36–1451). It can be clearly seen that it showed a single phase nature with hexagonal wurtzite structure. Refined value of the lattice parameters a and c of as prepared ZnO are found to be a = 3.253 Å and c = 5.246 Å, which are in good agreement with the standard data base [JCPDS, 36–1451]. Moreover, all diffraction peaks of the product show stronger peak intensities, indicating that the obtained ZnO nanoparticles have high crystallinity.

The crystallite size of the synthesized powder was estimated from x-ray lines broadening using Scherer’s equation^39^:


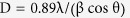


where λ is the wavelength (Cu Kα), β is the full width at the half-maximum and θ is the diffraction angle. The average grain size of ZnO nanoparticles was found ~26 nm.

**Figure 2:**
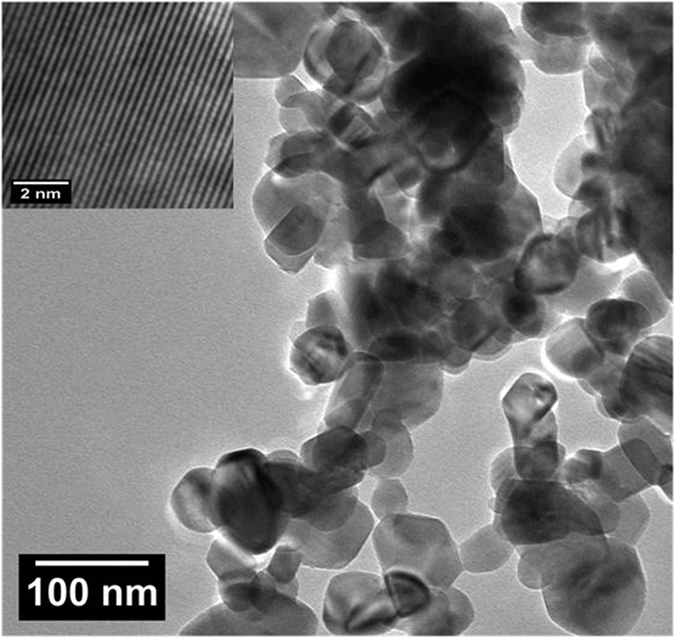
TEM micrograph of ZnO nanoparticles. The inset shows the corresponding HRTEM image.

The detailed morphology of ZnO nanostructures was investigated by TEM analysis. Figure 2 shows the TEM image of ZnO nanoparticles which are homogeneous and agglomerated with a particle size ~24 nm. Due to the uniform distribution of oxidized metal anions in the three-dimensional polymeric network structure, the agglomeration could be induced by densification resulting from the narrow space between particles. It is clear from the TEM micrographs that the particles have mixed morphology of spherical and elongated rod shape. Inset of Fig. 2 shows high resolution transmission electron microscopy (HRTEM) image of the ZnO nanoparticles. The HRTEM image shows clear lattice fringes indicating the highly crystalline ZnO wurtzite structure, and d spacing is well matched with the standard d values of ZnO.

**Figure 3:**
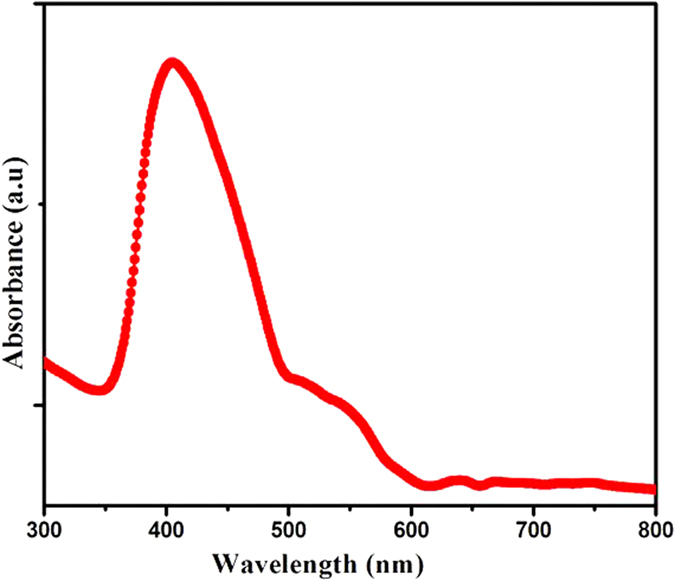
UV-Vis absorption spectrum of ZnO nanoparticles *Nigella sativa* seed extract.

UV–vis absorption spectrum as shown in Fig. 3, is carried out to evaluate the potential optical properties and to understand the electronic structure of the as-prepared ZnO nanoparticles. For the UV–vis absorption measurement, the as-prepared ZnO product is ultrasonically dispersed in absolute ethanol before examination, using absolute ethanol as the reference. The spectrum was corrected for the solvent contribution. The absorption spectrum of ZnO nanoparticles show well-defined exciton band at ~402 nm (calculated band gap of ~3.08 eV) which is red shifted by ~29 nm relative to the bulk exciton absorption (373 nm)^40^. The reason of the shifting of absorption band could be due to the oriented attachment of the nanoparticles by microwave irradiation, may lead to defect formation in the nanoparticles. The absorption in the visible range of wavelength implies that there exist more defect energy levels in the synthesized ZnO nanostructures that are due to the specific experimental synthesis conditions^41^. Similar observations for shifting of absorption bands of ZnO towards visible region were also reported earlier^42^. Surface area and surface defects play an important role in the functional properties of metal oxides, which affects the optical and electronic properties^43^, due to which the optical absorption shifts towards the visible region. One of the strategies adopted for tuning the band gap is to introduce intentional defects in the crystal lattice by which the electronic structure of ZnO can be altered. In this method, defects in ZnO nanoparticles might be generated by the microwave irradiation.

**Figure 4:**
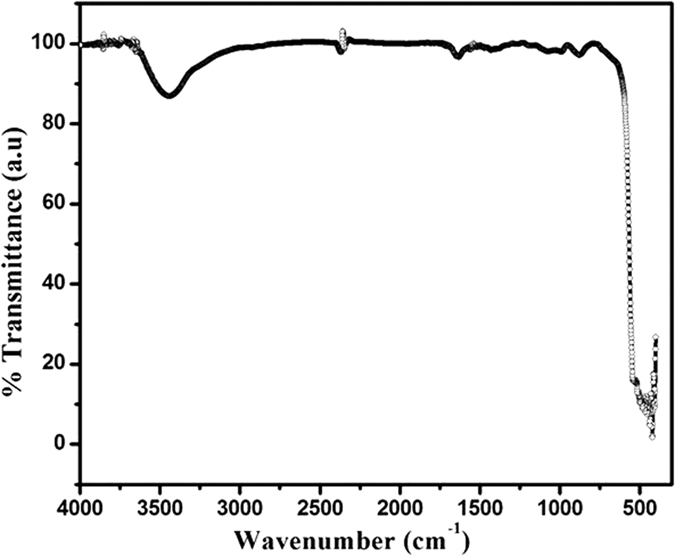
FT-IR spectrum of synthesized ZnO nanoparticles.

To study the change in Zn-O bonding, FTIR measurement of ZnO nanoparticles has been carried out. FTIR measurements were performed in the wave number range 4000–400 cm^−1^ using KBr method at Room temperature as shown in Fig. 4. The FTI spectrum shows main absorption bands near 3400 cm^−1^ represent O-H mode, band arising from the absorption of atmospheric CO_2_ on the metallic cations at 2300 cm^−1^ and 1400–1600 cm^−1^ are the C=O stretching mode. The absorption band at 431 cm^−1^ is the stretching mode of ZnO^44^ as shown in the inset of Fig. 4.

**Figure 5:**
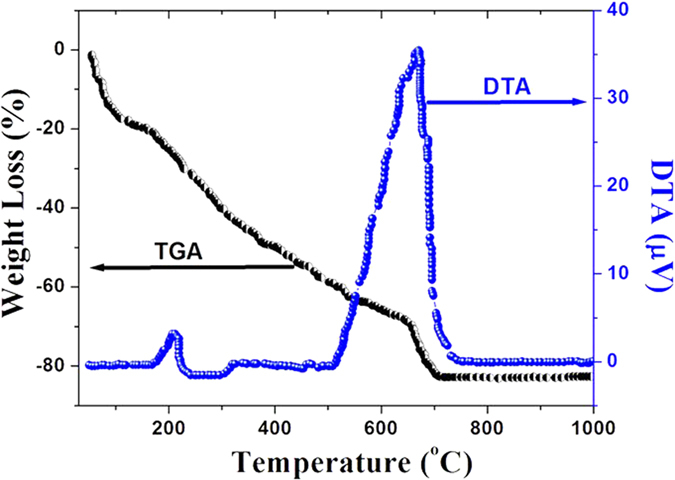
TGA/DTA curves for decomposition of bio-synthesized ZnO.

TGA and DTA surveys were conducted to investigate the formation of ZnO during the calcinations process. Figure 5 shows the TGA/DTA curves for decomposition of bio-synthesized ZnO. TGA showed a weight loss in two steps at around 220 and 650 °C and corresponding DTA showed two endothermic peaks at these temperatures. It is considered that the weight loss around 220 °C results from the removal of the bio-products by heating process to burn out the organics in air. Furthermore, the following weight loss up to 700 °C occurs due to the thorough decomposition of those charred products and the formation of ZnO. As seen from the differential curve, the peak at around 220 °C is due to the removal of absorbed water when the product is heated from room temperature to 220 °C. Upon prolongation of temperature, the major weight loss was attributed toward the loss of moisture and carbon. Herein the bio-synthesis of ZnO can be separated into two steps: one is the decomposition of bio-product, and the other is the crystallization of hexagonal ZnO by the complete removal of the bio-product during the calcinations process up to 700 °C.

### Minimum inhibitory concentration (MIC)

MICs of NS-ZnNPs was assessed against all pathogens (CV12472, CVO26, PAO1, *L. monocytogenes, E. coli*). The MICs of ZNP1 were found to be 512, 512, 128, 512, 256 μg/ml against CV12472, CVO26, PAO1, *L. monocytogenes, E. coli,* respectively. Concentrations below the MIC level were considered as Sub-MICs and were used throughout the study to assess the anti-QS and biofilm inhibitory properties of the NS-ZnNPs.

### QS (Violacein) inhibition assay

**Figure 6:**
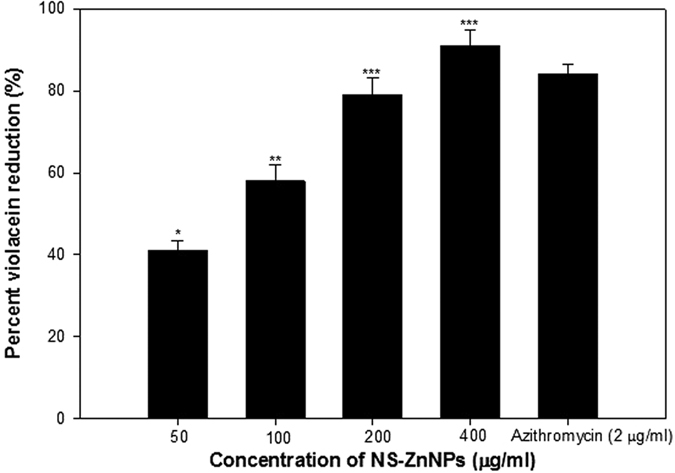
Quantitative assessment of violacein inhibition in CVO26 by sub-MICs of NS-ZnNPs. Data are represented as percentage of violacein inhibition. All of the data are presented as mean ± SD. *Significance at p ≤ 0.05, **significance at p ≤ 0.005, ***significance at p ≤ 0.001.

Anti-quorum sensing potential of NS-ZnNPs was screened using disc diffusion assay with bio-indicator strain *Chromobacterium violaceum* 12472, which produces the AHL-regulated violet-colored ‘violacein’ pigment. Concentration dependent violacein inhibition effect of NS-ZnNPs was recorded. Highest inhibition was recorded at 400 μg/ml followed by 200, 100 and 50 μg/ml while no pigment inhibition was observed at lower concentrations (Fig. 6). Azithromycin (2 μg/ml) was used as positive control.

Findings of the assay with *Chromobacterium violaceum* 12472 were further confirmed by colorimetric determination of violacein production in *Chromobacterium violaceum* CVO26. NS-ZnNPs at all tested concentration exhibited a statistically significant decrease in violacein content without inhibiting bacterial growth. At the concentration of 50 μg/ml NS-ZnNPs reduced violacein production up to 41% in comparison to untreated control (p ≤ 0.05). Dose dependent increase in the inhibitory activity was observed with increasing concentration of NS-ZnNPs to a maximum of 91% (p ≤ 0.001) at 400 μg/ml concentration of (Fig. 6).

### Inhibition of PAO1 virulence by sub-MICs of NS-ZnNPs

Effect of sub-MICs of synthesized NS-ZnNPs on the QS regulated virulence factors (elastase, total protease, pyocyanin production and alginate production) in PAO1 was assessed. Dose dependent decrease in the production of elastase (35–82%), total protease (20–77%) and pyocyanin production (48–93%) was recorded (Table 1). Alginate extracted from untreated and treated cultures of PAO1 was quantified. Gradual drop in alginate production was observed with increasing concentration of nanoparticle concentration. NS-ZnNPs demonstrated 20–73% reduction in alginate production of PAO1 at sub-MICs ranging from 10–80 μg/ml (Table 1).

### Effect on β-galactosidase activity

**Figure 7:**
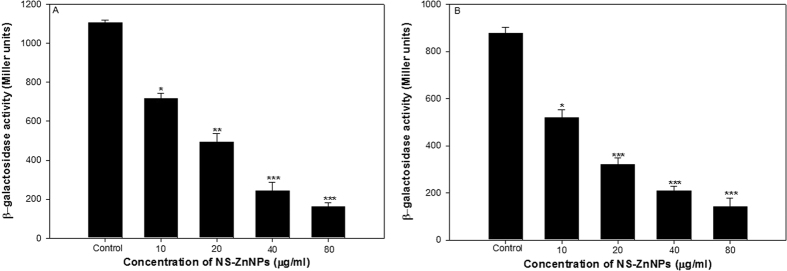
Effect of ceftazidime on las and pqs systems. (**A**) β-Galactosidase activity was measured in the *E. coli* MG4/pKDT17 with and without sub-MICs of NS-ZnNPs. (**B**) β-Galactosidase activity was measured in the *E. coli* pEAL08-2 with and without NS-ZnNPs.

Effect of 10–40 μg/ml concentrations of NS-ZnNPs on *lasB* and *pqsA* transcriptional activity was examined using β-galactosidase assay. NS-ZnNPs at 10, 20, 40 and 80 μg/ml concentrations significantly (p ≤ 0.001) reduced the *lasB* transcriptional activity by 35, 55, 78 and 85%, respectively. Moreover, 41–84% down regulation in *pqsA* was also recorded at concentration ranging from 10–80 μg/ml concentration of NS-ZnNPs (Fig. 7a,b). the reduction in transcriptional *pqsA* activity was significant at all tested concentrations.

### Swarming motility assay

**Figure 8:**
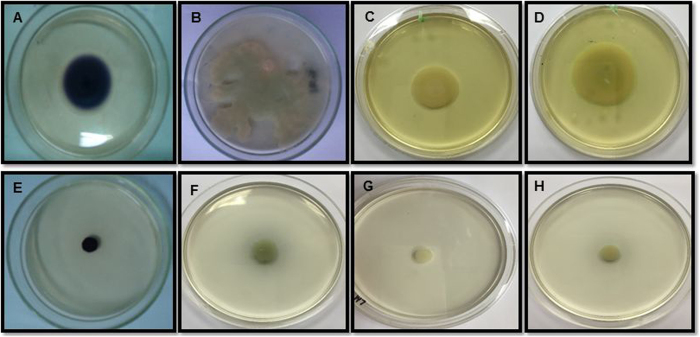
Effect of NS-ZnNPs on swarming motility of food pathogens. (**A**–**D**) NS-ZnNPs untreated and (**E**–**H**) treated (1/2 × MIC) plates of *C. violaceum*, PAO1, *L. monocytogenes, E. coli*, respectively.

The addition of sub-MICs of NS-ZnNPs showed a dose dependent decrease in the swarming migration of all the tested pathogens. The maximum inhibition in swarming migration was recorded at 1/2 × MIC against all the bacterial pathogens. The synthesized nanoparticle demonstrated 33–75%, 21–74%, 7–78% and 53–73% reduction in motility behavior of *C. violaceum, P. aeruginosa* PAO1, *E. coli* and *L. monocytogenes*, respectively, at concentrations ranging from 1/16 × MIC−1/2 × MIC (Table 2; Fig. 8).

### EPS quantification

**Figure 9:**
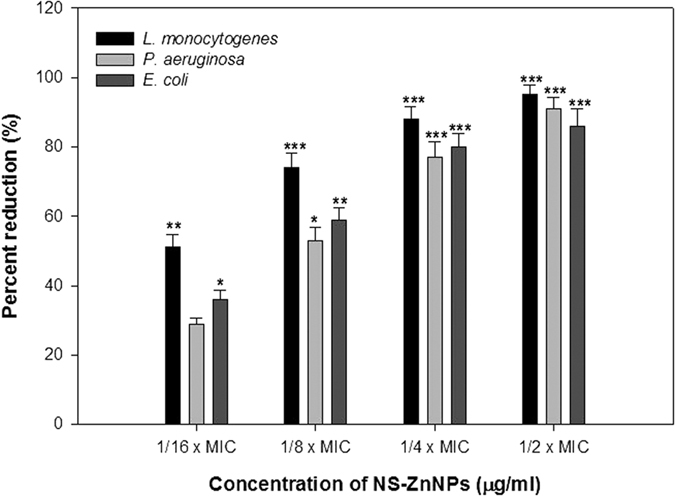
Quantitative analysis of EPS inhibition by measuring the absorbance at 490 nm. All of the data are presented as mean ± SD. *Significance at p ≤ 0.05, **significance at p ≤ 0.005, ***significance at p ≤ 0.001.

Spectrometric analysis of the extracted EPS revealed that the concentration of EPS decreased with increasing concentration of NS-ZnNPs. Statistically significant reduction in the EPS production was recorded at all sub-MICs tested (1/16 × MIC−1/2 × MIC). The biogenic nanoparticle (NS-ZnNPs) at 1/2 × MIC exhibited 95%, 91%, and 86% (p ≤ 0.001) decrease in EPS production of *L. monocytogenes, P. aeruginosa* PAO1, and *E. coli*, respectively (Fig. 9).

### Biofilm inhibition

**Figure 10:**
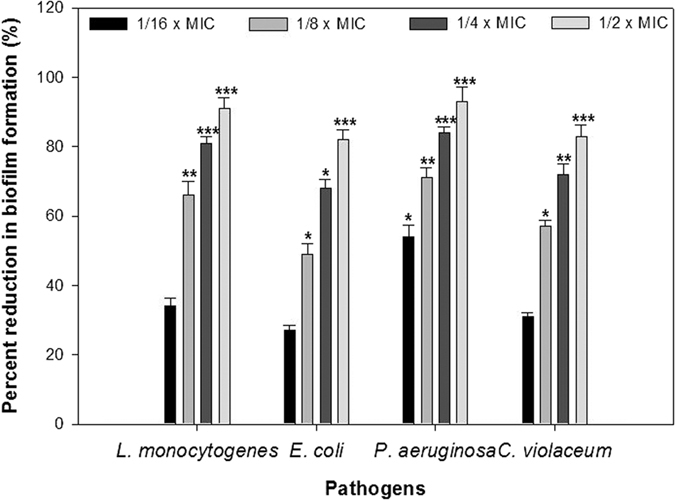
Quantitative measurement of biofilm inhibition as quantified by crystal violet staining and measuring absorbance at 470 nm. All of the data are presented as mean ± SD. *Significance at p ≤ 0.05, **significance at p ≤ 0.005, ***significance at p ≤ 0.001.

Microtiter plate assay for anti-biofilm activity of synthesized NS-ZnNPs showed a dose-dependent reduction in the biofilm biomass of test food pathogens. The data showed 34 ± 2.2, 66 ± 4.1, 81 ± 1.8 and 91 ± 3.2% inhibition of biofilm formation in *L. monocytogenes*; 54 ± 3.4, 71 ± 2.8, 84 ± 1.7 and 93 ± 4.3% in *P. aeruginosa* PAO1, 27 ± 1.5, 49 ± 3.1, 68 ± 2.6 and 82 ± 2.9% in *E. coli*; whereas 31 ± 1.2, 57 ± 1.6, 72 ± 3.1 and 83 ± 3.2% in *C. violaceum*, at their respective sub-MICs as compared to untreated control (Fig. 10). Statistically significantly impaired biofilm formation was recorded at all tested concentrations against all pathogens. The results of biofilm inhibition correlated positively with swarming and EPS reduction as these play an important role in adhesion and maturation of biofilms.

### *In-situ* visualization of biofilm inhibition

**Figure 11:**
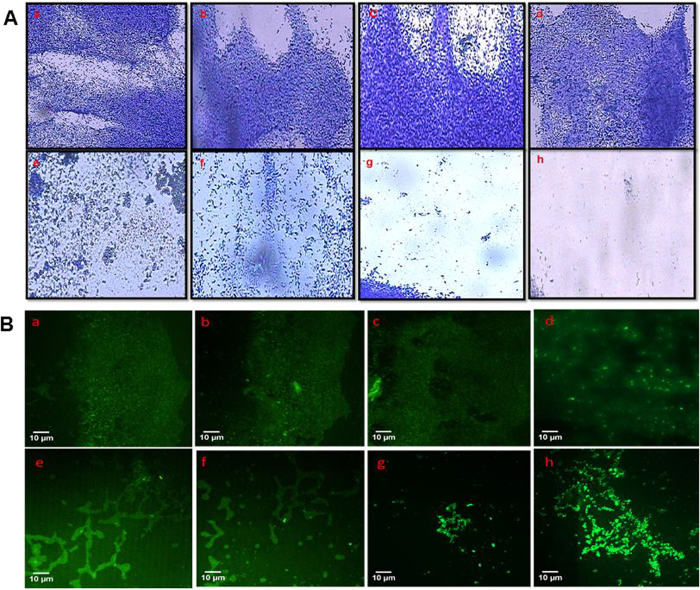
(**A**) Crystal violet stained light microscopic images of bacterial biofilms grown in the absence and presence of sub-MICs of NS-ZnNPs. (a–d) NS-ZnNPs untreated and (e–h) treated (1/2 × MIC) biofilms of *C. violaceum, E. coli, PAO1,* and *L. monocytogenes,* respectively. (**B**) Acridine orange stained confocal laser scanning microscopy (CLSM) images of (a–d) NS-ZnNPs untreated and (e–h) treated (1/2 × MIC) biofilms of *C. violaceum, E. coli, PAO1,* and *L. monocytogenes,* respectively. Scale bar 10 μm.

Untreated biofilms grown on glass coverslips showed a thick layer of biofilm, stained easily with CV, and visualized under the light microscope. However, NS-ZnNPs treated coverslips exhibited dose dependent impairment of biofilm formation of *L. monocytogenes, P. aeruginosa* PAO1, *C. violaceum* and *E. coli*. Results of microscopic analysis revealed the maximum level of reduction in number of microcolonies at the 1/2 × MIC against all tested food pathogens (Fig. 11A). Further, CLSM images also showed loose biofilm architecture and reduced biofilms of all foodborne pathogens after treatment with respective sub-inhibitory concentration in comparison to untreated control (Fig. 11B).

### Disruption of Mature Biofilm

**Figure 12:**
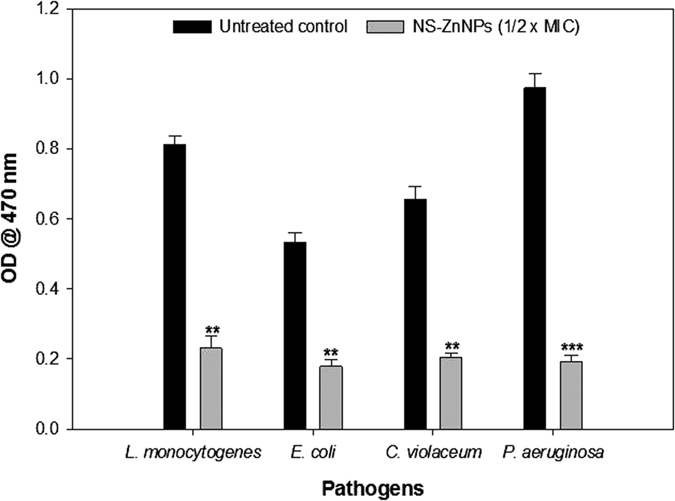
Quantitative measurement of dispersal of mature biofilms as quantified by crystal violet staining and measuring absorbance at 470 nm. All of the data are presented as mean ± SD. *Significance at p ≤ 0.05, **significance at p ≤ 0.005, ***significance at p ≤ 0.001.

Effect of NS-ZnNPson preformed biofilms of *L. monocytogenes, P. aeruginosa* PAO1, *C. violaceum*and *E. coli* was assessed by allowing biofilm formation for 16 h and inducing disruption for 8 h by addition of respective 1/2 × MIC of NS-ZnNPs. Crystal violet staining of disrupted biofilm revealed statistically significant reduction (p ≤ 0.005) of 72%, 66%, 68% and 78% in the preformed biofilms of *L. monocytogenes, E. coli, C. violaceum*, and *P. aeruginosa* PAO1, respectively (Fig. 12).

### Antibacterial activity of NS-ZnNPs in food model media

**Figure 13:**
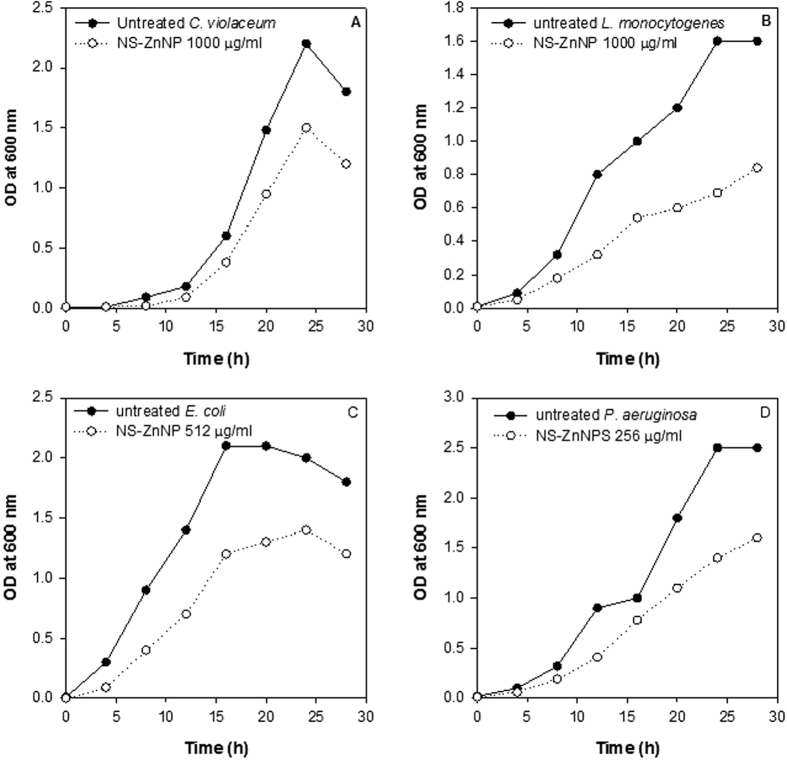
Antibacterial activity of NS-ZnNPs against test food pathogens in beef extract model media. Changes in absorbance, as an index of growth pattern of test bacteria, at respective NS-ZnNPs concentrations and as a function of time of incubation at 37 °C, are depicted in panels (**A**–**D**). Panel (s) (**A**): *C. violaceum* (Untreated) and treatment with 1000 μg/ml NS-ZnNPs; (**B**) *L. monocytogenes* (Untreated) and treatment with 1000 μg/ml NS-ZnNPs; (**C**) *E. coli* (Untreated) and treatment with 512 μg/ml NS-ZnNPs and (**D**) *P. aeruginosa* (Untreated) and treatment with 256 μg/ml NS-ZnNPs.

**Figure 14:**
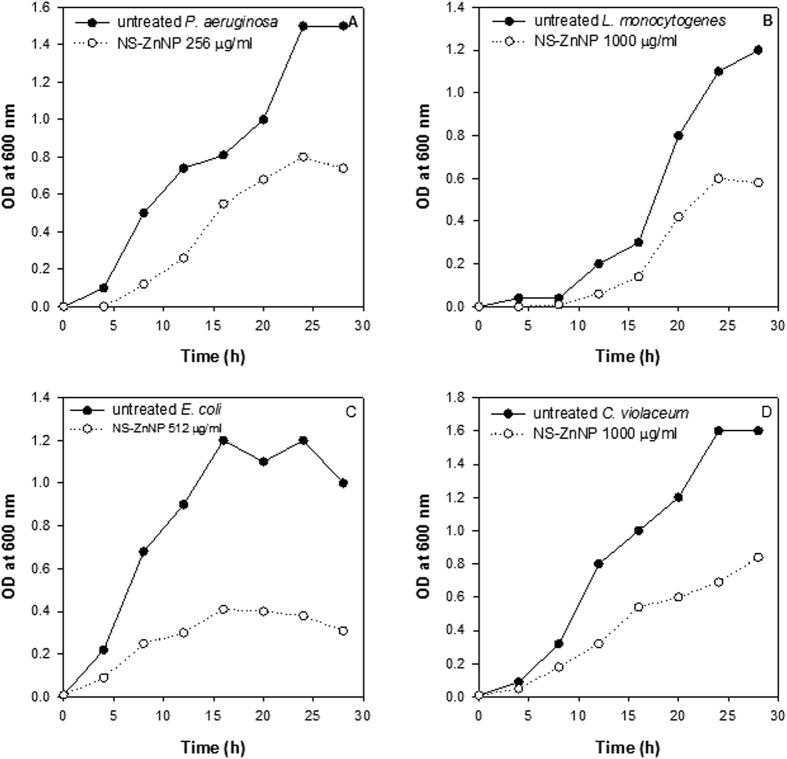
Antibacterial activity of NS-ZnNPs against test food pathogens in Lettuce leaf model media. Changes in absorbance, as an index of growth pattern of test bacteria, at respective NS-ZnNPs concentrations and as a function of time of incubation at 37 °C, are depicted in panels (**A**–**D**). Panel (s) (**A**) *P. aeruginosa* (Untreated) and treatment with 256 μg/ml NS-ZnNPs; (**B**) *L. monocytogenes* (Untreated) and treatment with 1000 μg/ml NS-ZnNPs; (**C**) *E. coli* (Untreated) and treatment with 512 μg/ml NS-ZnNPs and (**D**) *C. violaceum* (Untreated) and treatment with 1000 μg/ml NS-ZnNPs.

The NS-ZnNPs were evaluated for their antimicrobial activity against test pathogens in two food media models. The results shown in Figs 13 and 14 demonstrate significant growth inhibition of *L. monocytogenes, P. aeruginosa* PAO1, *C. violaceum* and *E. coli* at 1000, 256, 1000 and 512 μg/ml concentration of NS-ZnNPs, respectively. Figure 13(A–D) shows the reduction in growth rate for all the microbes tested after incubation with respective concentrations of NS-ZnONPs in beef extract media. Similarly, in the lettuce leaf model media significantly impaired growth of the test pathogens was recorded after treatment with NS-ZnNPs as compared to the untreated control (Fig. 14A–D). Antibacterial activity of the synthesized NS-ZnNPs was similar in both the model food mediums and it is envisaged that these zinc nanostructures have broad-spectrum antibacterial property.

## Discussion

Excessive food is lost due to quorum sensing regulated microbial spoilage and/or contamination of food by human pathogens^1^. Hence, disrupting the quorum-sensing circuit and biofilm formation can play a major role in controlling food spoilage and ensuring food safety.

Quorum sensing inhibitory activity of synthesized NS-ZnNPs was assessed using *Chromobacterium violaceum* CV12472 and CVO26 biosensor strains. Production of pigment violacein is regulated by CviIR-dependent quorum sensing system. Therefore, inhibition of pigment is suggestive of interference with AHL-regulated QS^45^. Concentration dependent reduction in pigment production (opaque, non-transparent halo zones) was observed around the disc loaded with NS-ZnNPs (Fig. 6). Further, for quantitative assessment of violacein inhibition an engineered biosensor strain *Chromobacterium violaceum* CVO26 was used. CVO26 does not produce its own its AHL but responds to exogenous AHL. The findings of colorimetric assay revealed significant reduction in violacein at all tested concentrations (50–400 μg/ml) of NS-ZnNPs. Our findings on QS inhibition in *Chromobacetrium violaceum* biosensor strain are well supported by those reported with silver nanowires (80% inhibition), mycofabricated silver nanoparticles (100% violacein inhibition) and AgCl-TiO_2_ nanoparticles^4^,^46^,^47^.

### Inhibition of PAO1 virulence by sub-MICs of NS-ZnNPs

To ascertain the QS inhibitory of NS-ZnNPs, effect of these nanoparticles was assessed on QS regulated virulence factors of *P. aeruginosa* PAO1. QS signaling in *Pseudomonas aeruginosa* is N-acyl homoserine lactones (AHLs) based and comprises of three QS systems namely, the *las, rhl* and *pqs* systems that regulate the production of virulence factors, including elastase, exoproteases, siderophores, exotoxins, rhamnolipid, pyocyanin, pyoverdin and participate in the development of biofilms^48^,^49^.

Proteases and elastase enzymes contribute greatly to the pathogenesis of *P. aeruginosa* by degrading host tissues enhancing the growth and invasiveness of the organism^50^. Sub-inhibitory concentrations of NS-ZnNPs inhibited elastase (35–82%) and protease (20–77%) significantly in dose dependent manner. Pyocyanin is a secondary metabolite of *P. aeruginosa* that causes severe toxic effects by damaging the neutrophil-mediated host defense^51^. Effect of NS-ZnNPs on pyocyanin was assayed and statistically significant decrease in production was recorded over untreated control (Table 1). *P. aeruginosa* biofilm matrix is composed of an expolysaccharide called alginate, its production is also known to be controlled by QS. The effect of sub-MICs of NS-ZnNPs on alginate production wasstudied and alginate production o was reduced significantly with increasing concentration of NS-ZnNPs. Results of alginate inhibition points towards successful disruption of biofilm matrix of *P. aeruginosa*. Our findings on inhibition of virulence factors find support from the observations on silver nanoparticles, Zinc nanoparticles, ceftazidime, menthol and caffeine^29^,^47^,^52^,^53^,^54^.

### Effect on β-galactosidase activity

Findings of the β-galactosidase activity showed that sub-MICs of NS-ZnNPs reduced both the elastase activity of PAO1 and the transcriptional activation of *lasB* in *E. coli*, indicating that synthesized NPs inhibits the *las* QS system. There is a positive co-relation between AHL concentration and *lasB–lacZ* expression as demonstrated by Pearson *et al*.^33^ Our findings correlate well with the above observations, as reduced β-galactosidase activity is indicative of reduced AHL levels and, therefore, reduced expression of the *lasB* gene. Since the production of pyocyanin production is regulated by *pqs* system, NS-ZnNPs inhibits the transcriptional activation of *pqsA* in *E. coli*, which indicates that NPs inhibits the *pqs* system. Results of the present assay demonstrates the broad-spectrum anti-QS property of these NPs as it inhibits the *las* and *pqs* systems of *P. aeruginosa*.

### Inhibition of swarming motility and EPS production

Flagella-driven swarming motility is a QS-dependent virulence function that play an important role in the initiation of cell/surface attachment during biofilm development^55^. Present investigation examined the ability of sub-inhibitory concentrations of synthesized NS-ZnNPs to impair the migration of test pathogens in a dose-dependent manner (Table 2, Fig. 8). Reduction in the swarming migration is indicative of the inhibition in the flagellar synthesis by the NS-ZnNPs. Thus, NS-ZnNPs indirectly effects the biofilm formation of all the target food pathogens in part by interfering with the ability of the pathogen to reach the substratum and subsequent biofilm formation by disturbing AHL-regulated QS system.

EPS plays a key role in the maintenance of biofilm architecture and provides increased resistance to the cells to antibiotics as well as to osmotic and oxidative stresses^48^. Therefore, any interference with EPS synthesis or production is bound to effect the biofilm architecture and bacteria in the biofilm mode of growth will become more susceptible to the administered antibiotics. Hence, resistance to drugs will also be reduced. In the present study, EPS was reduced significantly when bacterial pathogens were treated with sub-MICs of NS-ZnNPs (Fig. 9).

### Biofilm inhibition

Biofilm inhibitory properties of NS-ZnNPs were examined against foodborne pathogens *L. monocytogenes, E. coli, C. violaceum*, and *P. aeruginosa* PAO1. NS-ZnNPs reduced the biofilm biomass significantly in a dose-dependent manner (Fig. 10) without affecting the bacterial growth against all pathogens tested. Light microscopic images (Fig. 11A) revealed that NS-ZnNPs reduced the number of microcolonies during the biofilm formation of test bacterial pathogens. Therefore, it is envisaged that treatment of bacterial pathogens with NS-ZnNPs resulted in the formation of weak biofilms possibly by reducing the surface adhesion and subsequent microcolony formation. Further, CLSM analysis of NS-ZnNPs treated biofilms displayed poor and weakened architecture and reduced thickness than that of untreated biofilms of all pathogens tested (Fig. 11B). Our findings are agreement with the report on AgCl-TiO_2_ nanoparticles, where in, biofilm formation in *C. violaceum* was completely inhibited at 100 μg/ml^4^. In another study, silver nanowires arrested biofilm formation significantly at 4 mg/ml concentration without affecting viability of microbial cells^45^.

Although further research is necessary to unearth the plausible mechanism of biofilm inhibition by the ZnO nanoparticles. Considering *P. aeruginosa* PAO1 as a model organism, Lee *et al*.^56^ reported that two-component response regulator CzcR is required for the inhibition of biofilm and pyocyanin. They demonstrated that ZnO nanoparticles inhibit pyocyanin production and biofilm formation via the *czcRS* system and *rhlR* quorum sensing system and also repress the production of the PQS autoinducer. In addition, increased cell surface hydrophobicity after treatment with ZnO NPs was another major reason for the impaired biofilm formation in PAO1^56^. Our findings on the inhibition of pyocyanin and biofilm formation by sub-MICs of NS-ZnNPs, correlates well with the findings of Lee *et al*.^56^ Therefore, it is predicted that the synthesized NS-ZnNPs inhibit biofilm via response regulator CzcR and increased hydrophobicity in *P. aeruginosa* PAO1.

### Disruption of mature biofilm

Since quorum sensing is helps in the maturation of biofilms, therefore, interference with QS mechanism is deemed to impair maturation of biofilms. Figure 12 shows significant reduction in preformed biofilms of all tested bacterial pathogens upon treatment with sub-inhibitory concentrations of NS-ZnNPs. Our results are in accordance with the previous report where Silver(I) complexes with phthalazine and quinazoline demonstrated marked ability to disrupt mature biofilms of *P. aeruginosa*^57^. Extract of *Capparis spinosa* also showed significant reduction in the preformed biofilms of *Escherichia coli, Proteus mirabilis, Serratia marcescens* and PAO1^58^.

### Antibacterial activity of NS-ZnNPs in food model media

To assess the effectiveness of the synthesized NS-ZnNPs as antimicrobial agents in food. We studied the antibacterial action of these nanostructures in two food model media namely, lettuce leaf model media and beef extract media. The NS-ZnNPs demonstrated significant reduction in the growth pattern of the tested pathogens in both the food models as shown in Figs 13 and 14. Our results find support from the observations made with natural products, alone or in combination with other preservative, against food-borne pathogens when applied to meat^38^,^59^, vegetable^38^,^60^ or milk^61^. Results obtained in food model media are important as they may provide the leads for further studies on food directly and help in the optimization of the test agents before final application. These model media are better tool than the commonly used standard laboratory media.

## Conclusion

In conclusion, our result demonstrates the development of a rapid, cost-effective, eco-friendly and safe method of zinc oxide nanoparticle synthesis exploiting the reducing and capping potential of *Nigella sativa* (black seed). Study further highlights the broad-spectrum attenuation of AHL mediated quorum sensing and QS regulated functions of *C. violaceum* and *P. aeruginosa* by synthesized NS-ZnNPs. Biogenic nanoparticles (NS-ZnNPs) impaired the biofilm formation of four food pathogens *viz. L. monocytogenes, P. aeruginosa* PAO1, and *E. coli* considerably. Vital factors like swarming motility and EPS production, contributing in the initial attachment and maturation of biofilm were also reduced significantly. Moreover, significant reduction in preformed biofilms of all tested bacterial pathogens upon treatment with sub-inhibitory concentrations of NS-ZnNPs is a key finding. This is probably the first report on the broad-spectrum inhibition of QS and biofilm by biogenic Zinc oxide nanoparticles. The study projects the synthesized Zinc nanostructures to be a potential QS and biofilm inhibitor that can not only be exploited as an antipathogenic but nontoxic bioactive material that can be used as food packaging material and/or as food preservative.

## Additional Information

**How to cite this article**: Al-Shabib, N. A. *et al*. Biogenic synthesis of Zinc oxide nanostructures from *Nigella sativa* seed: Prospective role as food packaging material inhibiting broad-spectrum quorum sensing and biofilm. *Sci. Rep.* **6**, 36761; doi: 10.1038/srep36761 (2016).

**Publisher's note:** Springer Nature remains neutral with regard to jurisdictional claims in published maps and institutional affiliations.

## Figures and Tables

**Table 1:** Effect of sub-MICs of NS-ZnNPs on quorum sensing regulated virulence factors in *P. aeruginosa* PAO1.

NS-ZnNPs concentration (μg/ml)	Elastase activity^a^	Total protease^b^	Pyocyanin production^c^	Chitinase activity^d^	Alginate production^e^
Control	0.188 ± 0.025	0.936 ± 0.041	5.5 ± 0.25	0.159 ± 0.008	0.809 ± 0.037
10	0. 123 ± 0.015 (35)	0.751 ± 0.021 (20)	2.9 ± 0.2 (48)*	0.081 ± 0.004 (49)*	0.647 ± 0.015 (20)
20	0.105 ± 0.028 (44)*	0.591 ± 0.030 (37)	1.4 ± 0.13 (74.5)***	0.051 ± 0.005 (68)**	0.471 ± 0.018 (42)*
40	0.062 ± 0.007 (67)**	0.348 ± 0.014 (63)**	0.9 ± 0.052 (84)***	0.045 ± 0.004 (72)**	0.301 ± 0.029 (63)*
80	0.034 ± 0.003 (82)***	0.215 ± 0.012 (77)***	0.4 ± 0.024 (93)***	0.028 ± 0.008 (82)***	0.214 ± 0.024 (73)**
Azithromycin (2 μg/ml)	0.043 ± 0.04	0.289 ± 0.031	0.31 ± 0.007	0.034 ± 0.027	0.203 ± 0.018

The data represents mean values of three independent experiments. ^*^Significance at p ≤ 0.05, ^**^significance at p ≤ 0.005, ^***^significance at p ≤ 0.001. Values in the parentheses indicate percent reduction over control.

^a^Elastase activity is expressed as the absorbance at OD_495_.

^b^Total protease activity is expressed as the absorbance at OD_600_.

^c^Pyocyanin concentrations were expressed as micrograms of pyocyanin produced per microgram of total protein.

^d^Chitinase activity is expressed as the absorbance at OD_570_.

**Table 2:** Effect of NS-ZnNPs at different concentrations on swarming motility of bacterial pathogens.

Bacterial pathogens	Diameter of swarming migration (mm)				
	Control	1/16 × MIC	1/8 × MIC	1/4 × MIC	1/2 × MIC
*C. violaceum*	48 ± 2.1	32 ± 1.3	21 ± 0.7	18 ± 1.6	08 ± 0.6
*P. aeruginosa*	51 ± 1.4	40 ± 0.5	26 ± 1.1	15 ± 0.8	11 ± 1.3
*E. coli*	41 ± 1.7	38 ± 2.4	31 ± 0.4	19 ± 0.8	09 ± 0.5
*L. monocytogenes*	30 ± 0.8	14 ± 1	11 ± 0.5	10 ± 0.7	07 ± 1
