# Erratum: Biogenic synthesis of Zinc oxide nanostructures from *Nigella sativa* seed: Prospective role as food packaging material inhibiting broad-spectrum quorum sensing and biofilm

**DOI:** 10.1038/srep42266

**Published:** 2017-02-09

**Authors:** Nasser A. Al-Shabib, Fohad Mabood Husain, Faheem Ahmed, Rais Ahmad Khan, Iqbal Ahmad, Edreese Alsharaeh, Mohd Shahnawaz Khan, Afzal Hussain, Md Tabish Rehman, Mohammad Yusuf, Iftekhar Hassan, Javed Masood Khan, Ghulam Md Ashraf, Ali Alsalme, Mohamed F. Al-Ajmi, Vadim V. Tarasov, Gjumrakch Aliev

Scientific Reports 6: Article number: 3676110.1038/srep36761; published online: 12 05 2016; updated: 02 09 2017

The original version of this Article contained a typographical error in the spelling of Ali Alsalme, which was incorrectly given as Ali Mohammed Alsalme.

In addition, Mohamed F. Al-Ajmi was incorrectly affiliated to “Protein Research Chair, Department of Biochemistry, College of Science, King Saud University, Riyadh-11451, Kingdom of Saudi Arabia”. The correct affiliation for this Author is listed below:

Department of Pharmacognosy, College of Pharmacy, King Saud University, Riyadh-11451, Kingdom of Saudi Arabia.

In addition, there was a typographical error in the legend of Figure 7, where:

“Effect of ceftazidime on las and pqs systems”.

Now reads:

“Effect of NS-ZnNPs on las and pqs systems”.

These errors have now been corrected in the HTML and PDF versions of this Article.

